# Uric Acid Variability as a Predictive Marker of Newly Developed Cardiovascular Events in Type 2 Diabetes

**DOI:** 10.3389/fcvm.2021.775753

**Published:** 2021-12-02

**Authors:** Hae Kyung Kim, Minyoung Lee, Yong-ho Lee, Byung-Wan Lee, Bong-Soo Cha, Eun Seok Kang

**Affiliations:** ^1^Department of Internal Medicine, Yonsei University College of Medicine, Seoul, South Korea; ^2^Institute of Endocrine Research, Yonsei University College of Medicine, Seoul, South Korea

**Keywords:** cardiovascular disease, percutaneous coronary intervention, diabetes mellitus type 2, uric acid, variability

## Abstract

**Background:** Cardiovascular disease (CVD) is associated with morbidity and mortality in patients with type 2 diabetes mellitus (T2D). However, the role of serum uric acid as a risk factor for developing cardiovascular disease is controversial. This study investigated whether uric acid variability was associated with new-onset symptomatic CVD in patients with T2D, requiring percutaneous coronary intervention.

**Methods:** A total of 1,071 patients were enrolled in this retrospective cross-sectional study after propensity score matching. Patients with T2D and new-onset symptomatic CVD who received percutaneous coronary intervention for the first time, and with at least three consecutive 6-monthly measurements of serum uric acid were recruited from Severance Hospital between January 2015 and December 2019. Uric acid variability was measured by average successive variability (ASV) and analyzed to evaluate a possible correlation with the risk of developing CVD.

**Results:** The patients were divided into quartiles based on the uric acid variability. Patients in the highest quartile were older and presented lower renal function and a higher mortality from CVD. There was a linear association between a high uric acid variability and the development of CVD. Compared to the lowest quartile, patients in the higher quartiles had a higher risk of CVD [quartile 3: adjusted odds ratio (aOR) = 1.76; 95% confidence interval (CI), 1.20–2.82; *P* = 0.019; quartile 4 aOR = 2.89; 95% CI, 1.74–4.80; *P* < 0.001].

**Conclusion:** High uric acid variability is independently associated with an increased risk of new-onset symptomatic CVD requiring percutaneous coronary intervention in patients with T2D. Thus, maintaining serum uric acid in a narrow range by prescribing effective medications is essential to prevent new-onset CVD in patients with T2D. Nonetheless, the potential use of uric acid variability as a predictive marker of CVD in patients with T2D needs further validation.

## Introduction

Type 2 diabetes mellitus (T2D) is a major risk factor for developing cardiovascular diseases (CVD) (1) and is closely associated with morbidity and mortality (2). A previous population-based large cohort study has shown that patients with T2D tend to develop CVD, especially acute myocardial infarction ~15 years earlier than patients without T2D (3). In addition, a meta-analysis of prospective studies found that T2D increased the risk of new-onset ischemic heart disease two-folds (4). Therefore, detecting the signs of CVD and managing its risk factors in patients with T2D is essential.

Uric acid is an antioxidant, particularly in the extracellular environment (5). However, in the cytoplasm or the acidic or hydrophobic milieu of atherosclerotic plaques, uric acid is converted to pro-oxidant agents, promoting oxidative stress, which can accelerate the development of CVD (6). Epidemiological studies suggested a positive relationship between elevated serum uric acid (SUA) levels and the risk of CVD, metabolic syndrome, insulin resistance, diabetes, and chronic kidney disease (7). High SUA is considered risk factor for developing CVD, especially in patients with established or at high risk of coronary heart diseases (8). Available meta-analyses have not reached consensus regarding the role of uric acid as an independent risk factor for CVD (9, 10). This discordance may be because most studies measured serum uric acid only once in the general population without diabetes. Moreover, although hyperuricemia is a risk factor for CVD, urate-lowering drugs do not improve CVD outcomes, including a composite of cardiovascular death, non-fatal myocardial infarction, non-fatal stroke and unstable angina requiring urgent revascularization (11).

The concept of long-term variability, defined as the variability in clinical measures outside the recommended range, is currently a well-accepted. Long-term glycemic variability correlates with an increased risk of macro- and microvascular complications in patients with T2D (12) and increased variability in blood pressure, lipid parameters, body weight and heart rate contributes to the development of CVD (13–15). In addition, a higher variability in SUA measured by standard deviations (SD) was associated with coronary heart disease and all-cause mortality in male patients without diabetes (16) and increased the risk of developing future cardiovascular events in patients who underwent percutaneous coronary interventions (17). Although uric acid is a shared determinant for the development of T2D and CVD, no studies have assessed the correlation between uric acid variability and CVD in patients with T2D.

This study investigated whether uric acid variability can predict the risk of development of new-onset symptomatic CVD in patients with T2D. By determining the importance of uric acid variability relative to CVD with view to provide evidence-based guidance to clinicians for preventing CVD by measuring and managing SUA levels in patients with T2D.

## Methods

### Study Population

This retrospective cross-sectional observational study included patients, aged ≥19 years, with T2D and at least three consecutive measurements of 6-montly SUA as well as simultaneously evaluated blood and urinary markers at the Severance Hospital, between January 2015 and December 2019. T2D was defined as follows: (1) the use of insulin or oral hypoglycemic agents or (2) HbA1c ≥ 6.5% (47.5 mmol/mol) according to the Practical Guidelines of the Korean Diabetes Association (18). The exclusion criteria were: age <19 years, type 1 diabetes, pregnancy, active cancer, renal replacement therapy (including renal transplantation and dialysis), and previously diagnosed CVD with percutaneous coronary intervention identified by International Classification of Diseases (ICD) codes. Patients were divided according to the presence of new-onset symptomatic CVD. The diagnosis of new-onset symptomatic CVD included participants who underwent PCI either with coronary stenting or balloon angioplasty with confirmation of positive results of ischemic change on electrocardiogram, and by identifying ICD codes, as well as reviewing electronic medical records. Among 15,833 eligible participants, 357 patients were diagnosed with new-onset symptomatic CVD (CVD group) (Supplementary Figure 1).

Demographic data, including age, sex, body weight, height, waist circumference, blood pressure, duration of diabetes, and use of medications, were obtained. Body mass index (BMI) was calculated as body weight divided by height squared (kg/m^2^). Cigarette smoking was self-reported, and classified as ever smokers or not. Hypertension was defined as systolic blood pressure of ≥140 mmHg or diastolic blood pressure of ≥90 mmHg, or the current use of antihypertensive medications according to 2005 Guidelines of the American Heart Association and the National Heart, Lung, and Blood Institute for Asian populations (19). Dyslipidemia was diagnosed according to the guidelines established by the National Cholesterol Education Program Adult Treatment Panel III (20). Angiographic data included indications for PCI, target vessel and the number of lesions treated. Cardiovascular mortality was defined on a case-by-case basis by reviewing electronic medical records to identify a causal relationship between CVD and mortality. The history of cerebrovascular disease was confirmed by analyzing hospital records.

### Propensity Score Matching

Given the retrospective and non-randomized nature of the study, the CVD groups and non-CVD group were compared to assess the risk of CVD according to uric acid variability. A propensity score, defined as the conditional probability of receiving treatment based on covariates was used to balance the covariates in the two groups, thereby reducing selection bias (21). Propensity scores were estimated using a non-parsimonious multiple logistic regression model. These scores were calculated using all potential confounders to minimize selection bias between CVD and non-CVD groups. The risk of CVD according to quartiles of uric acid variability were assessed using, 1:2 propensity score matching (PSM). Before PSM, 357 patients in the CVD group and 15,476 patients in the non-CVD group met the eligibility criteria. After PSM, 357 patients from CVD group were matched to 714 patients from the non-CVD group, using the nearest neighbor matching algorithm with a caliper of 0.2. The covariate balance between the matched groups was examined.

### Assessment of Uric Acid Variability

SUA was measured at least three times, with a minimum interval of 6 months prior to the index year of CVD event. Average successive variability (ASV) was used because of the non-normal distribution of SUA levels. ASV was defined as the average of absolute difference between successive values and was calculated using the formula |FPG_1_-FPG_2_| + |FPG_2_ – FPG_3_|+ ··· + |FPG_n−1_-FPG_n_|/*n*−1 where n is the number of uric acid measurements. The patients were grouped into quartiles based on the uric acid variability. Previous studies used ASV to measure the variability of other biochemical markers related to cardiovascular outcomes and mortality, including blood pressure, glucose, cholesterol, and BMI (15).

### Measurement of Metabolic Parameters

Following an overnight fast (≥8 h), morning spot urine samples were collected, and albumin, protein, and creatinine levels were quantified. Blood samples were collected, and the complete blood count, lipid profile, fasting glucose, insulin, C-peptide, HbA1c, and creatinine were measured. HbA1c was measured by immunoassay using an Integra 800 CTS analyzer (Roche, Hercules, CA, USA). Serum glucose and creatinine were quantified using a Hitachi 7,600 analyzer (Hitachi Ltd.). Serum creatinine levels were determined using the compensated kinetic Jaffe method (Clinimate CRE, Sekisui Medical Co., Ltd., Japan), in which creatinine concentration was standardized to isotope dilution-mass spectrometry. Low-density lipoprotein cholesterol (LDL-C) levels were calculated using the Friedewald equation without actual LDL-C measurements (22); however, this equation was not used if the patient's triglyceride level exceeded 400 mg/dL. Pancreatic β-cell function and insulin sensitivity in on-insulin users were assessed using the following indices: homeostasis model assessment-β-cell (HOMA-β) = [20 × fasting insulin (μIU/mL)]/[fasting plasma glucose (mmol/L)−3.5] and homeostasis model assessment-insulin resistance (HOMA-IR) = [fasting insulin (μIU/mL) × fasting plasma glucose (mmol)]/22.5 (23). Estimated GFR (eGFR) was calculated using the Chronic Kidney Disease Epidemiology Collaboration (CKD-EPI) equation (24). Urinary albumin levels were adjusted as urinary albumin to creatinine ratio (u-ACR) to minimize the influence of kidney function. Albuminuria was defined as u-ACR ≥30 mg/g according to the Kidney Disease: Improving Global Outcomes recommendations (25).

### Statistical Analysis

The data are presented as mean ± SD for normally distributed continuous variables and as medians (interquartile range) for non-normally distributed continuous variables. Categorical variables were expressed as numbers and percentages. The baseline characteristics of the CVD group and non-CVD group were compared based on the quartiles of uric acid variability. Participants' characteristics according to quartiles of baseline uric acid variability were analyzed using one-way analysis of variance (ANOVA) or Kruskal-Wallis test to compare continuous variables, and Chi-square test to compare categorical variables, followed by *post-hoc* analyses using Bonferroni procedure for ANOVA and Dunn procedure for Kruskal-Wallis test. The normality of continuous variables was evaluated using the Shapiro-Wilk test. Correlations between ASV and other parameters were evaluated using Pearson's correlation analysis. The independent association between ASV and CVD after adjusting for age, sex, baseline SUA, hypertension, statin use, eGFR, HbA1c, and duration of diabetes was evaluated by multiple logistic regression analysis. Survival was assessed using the Kaplan-Meier methods and Cox proportional hazard regression model and was compared using the log-rank test. To assess the predictive accuracy of uric acid variability by ASV for CVD adjusted by multiple variables, receiver operating characteristic (ROC) curves (AUC) was calculated. Differences between ROC curves were evaluated according to Delong et al. (26) and ability of SD and ASV for CVD was calculated using continuous net reclassification index (NRI) and integrated discrimination improvement (IDI) (27). Statistical analyses were performed using SPSS statistical software version 25.0 for Windows (IBM, Armonk, NY, USA). Statistical significance was set at *P* < 0.05.

## Results

### Baseline Characteristics of the Study Population

A total of 1,071 patients (357 in CVD group, and 714 in non-CVD group) were enrolled in this study. The patients were divided into quartiles according to the uric acid variability (Table 1). The mean age and duration of diabetes were 69 and 15 years, respectively. The study population consisted of 67.9% male and 36.7% smokers. The mean BMI and baseline SUA were 25.0 ± 5.6 kg/m^2^ and 5.18 ± 1.53 mg/dL, respectively. The ASV of quartiles of uric acid were 0.23 ± 0.09 (quartile 1), 0.47 ± 0.06 (quartile 2), 0.77 ± 0.13 (Quartile 3), and 1.66 ± 0.80 (Quartile 4). Patients in the highest quartile were older and presented higher baseline SUA, lower high-density lipoprotein (HDL) cholesterol, higher creatinine, lower eGFR and higher mortality from CVD and hypertension. Blood pressure, waist circumference, lipid profile, except for HDL, u-ACR, the use of angiotensin converting enzyme (ACE) inhibitor and angiotensin II receptor blocker (ARB), angiographic data and the prevalence of dyslipidemia and cerebrovascular disease were not significantly different across quartiles. The use of insulin and uric acid lowering agents was more common in patients in quartiles 3 and 4. In contrast, the use of metformin and statin was more common in the patient in quartiles 1 and 2. Mean fasting glucose and HbA1c were 155 mg/dL and 7.2%, respectively, but did not exhibit significant differences between the quartiles, including HOMA-β and HOMA-IR.

**Table 1:** Baseline characteristics according to quartiles of uric acid variability.

	**All subjects** **(*N* = 1,071)**	**Quartile 1** **(*N* = 265)**	**Quartile 2** **(*N* = 230)**	**Quartile 3** **(*N* = 308)**	**Quartile 4** **(*N* = 268)**	***P*-value**
Uric acid A (mg/dL)	5.18 ± 1.53	4.7 ± 1.3	5.1 ± 1.3	5.2 ± 1.3	5.6 ± 2.0	**<0.01**
Uric acid B (mg/dL)	5.36 ± 1.61	4.8 ± 1.3	5.2 ± 1.4	5.4 ± 1.3	6.0 ± 2.1	**<0.01**
Uric acid C (mg/dL)	5.24 ± 1.62	4.8 ± 1.3	5.2 ± 1.4	5.3 ± 1.3	5.7 ± 2.1	**<0.01**
Uric acid variability (mg/dL)	0.79 ± 0.67	0.23 ± 0.09	0.47 ± 0.06	0.77 ± 0.13	1.66 ± 0.80	**<0.01**
Variability range	0.40–1.05	0.001–0.40	0.40–0.60	0.6–1.05	1.05–6.30	**<0.01**
Total follow up (months)	12 (11–13)	12 (11–13)	11 (11–13)	12 (11–13)	12 (11–13)	0.82
Age (years)	69 ± 9.8	68 ± 10	68 ± 10	68 ± 9.2	70 ± 9.8	**0.032**
Male (*n*, %)	727 (67.9)	171 (64.5)	157 (68.3)	208 (67.5)	191 (71.3)	0.42
BMI (kg/m^2^)	25.0 ± 5.6	25 ± 3.8	25 ± 3.1	26 ± 8.7	24 ± 3.9	0.18
Waist circumference (cm)	91 (84–96)	91 (83–97)	91 (85–95)	92 (84–96)	89 (83–97)	0.93
Systolic blood pressure (mmHg)	127 ± 49	123 ± 16	125 ± 15	130 ± 88	128 ± 19	0.74
Diastolic blood pressure (mmHg)	70 ± 13	70 ± 10	70 ± 14	70 ± 13	73 ± 15	0.13
Ever-smoked (*n*, %)	393 (36.7)	90 (33.9)	82 (35.7)	121 (39.3)	100 (37.3)	0.79
Duration of diabetes (years)	15.5 ± 9.80	14.7 ± 9.15	15.8 ± 9.05	15.9 ± 10.7	15.4 ± 9.92	0.58
Fasting glucose (mg/dL)	155 ± 63	157 ± 65	156 ± 61	151 ± 59	155 ± 69	0.68
HbA1c (%)	7.2 ± 1.3	7.2 ± 1.1	7.3 ± 1.3	7.2 ± 1.3	7.2 ± 1.4	0.91
HOMA-β	31.9 (17.5–61.0)	30 (13–60)	28 (20–60)	37 (17–63)	41 (22–66)	0.65
HOMA-IR	3.1 (1.7–5.2)	3.1 (1.6–6.2)	3.3 (1.8–4.7)	3.1 (1.9–4.5)	3.1 (1.2–5.7)	0.90
Total cholesterol (mg/dL)	149.9 ± 38	150 ± 33	154 ± 38	147 ± 43	147 ± 43	0.22
HDL cholesterol (mg/dL)	46 ± 13	47 ± 12	45 ± 11	46 ± 13	44 ± 14	**0.029**
LDL cholesterol (mg/dL)	77 ± 33	76 ± 29	82 ± 33	75 ± 31	76 ± 38	0.14
Triglyceride (mg/dL)	121 (89–168)	113 (87–160)	153 (128–175)	119 (88–169)	129 (92–180)	0.15
Creatinine (mg/dL)	0.95 ± 0.30	0.88 ± 0.25	0.93 ± 0.29	0.94 ± 0.26	1.04 ± 0.35	**<0.01**
eGFR(CKD-EPI) (mL/min/1.73 m^2^)	80.1 ± 23.3	83.5 ± 19.2	81.7 ± 23.0	80.8 ± 21.8	74.9 ± 27.7	**<0.01**
eGFR <60 ml/min/1.73 m^2^	219 (20.4)	35 (13.2)	43 (18.7)	54 (17.5)	87 (32.5)	**<0.01**
u-ACR (U/g creatinine)	44.6 (12.1–177)	16 (8.57–102)	50 (10.2–191)	73.4 (12.5–195)	53.6 (19.8–268)	0.30
**Indication for PCI (** ***n*** **, %)**						
Stable angina	126 (11.8)	30 (11.3)	27 (11.7)	41 (13.3)	28 (10.4)	0.75
UA/NSTEMI	88 (8.2)	21 (7.9)	18 (7.8)	28 (9.1)	21 (7.8)	0.93
STEMI	17 (1.6)	3 (1.1)	3 (1.3)	6 (1.9)	5 (1.9)	0.84
**Target vessel (** ***n*** **, %)**						
LAD	132 (12.3)	28 (10.6)	33 (14.3)	47 (15.3)	24 (9.0)	0.075
LCX	43 (4.0)	10 (3.8)	13 (5.7)	7 (2.3)	13 (4.9)	0.21
RCA	79 (7.4)	20 (7.5)	13 (5.7)	27 (8.8)	19 (7.1)	0.59
LM	10 (0.9)	2 (0.8)	0 (0)	3 (1.0)	5 (1.9)	0.19
**Number of lesions treated (** ***n*** **, %)**						
1	83 (7.7)	18 (6.8)	18 (7.8)	26 (8.4)	21 (7.8)	0.91
2	71 (6.6)	14 (5.3)	13 (5.7)	28 (9.1)	16 (6.0)	0.23
≥3	84 (7.8)	23 (8.7)	18 (7.8)	21 (6.8)	22 (8.2)	0.86
**CV mortality (** ***n*** **, %)**	19 (1.77)	0 (0)	3 (1.30)	5 (1.62)	11 (4.10)	**0.042**
**Comorbidity (** ***n*** **, %)**						
Hypertension	844 (78.8)	193 (72.8)	174 (75.7)	256 (83.1)	221 (82.5)	**0.006**
Dyslipidemia	961 (92.0)	240 (91.6)	206 (92.4)	279 (93.0)	236 (91.1)	0.85
Cerebrovascular disease	150 (14.0)	27 (10.2)	36 (15.7)	41 (13.3)	46 (17.2)	0.11
**Antidiabetic medication (** ***n*** **, %)**						
Metformin	763 (71.2)	200 (75.5)	166 (72.2)	231 (75.0)	166 (61.9)	**0.001**
Sulfonylurea	347 (32.4)	80 (30.2)	74 (32.2)	107 (34.7)	86 (32.1)	0.71
DPP-4 inhibitor	583 (54.4)	142 (53.6)	122 (53.0)	172 (55.8)	147 (54.9)	0.91
Thiazolidinedione	141 (13.2)	44 (16.6)	34 (14.8)	36 (11.7)	27 (10.1)	0.11
SGLT2 inhibitor	88 (8.2)	26 (9.8)	20 (8.7)	25 (8.1)	17 (6.3)	0.53
GLP-1 receptor analog	14 (1.3)	4 (1.5)	4 (1.7)	4 (1.3)	2 (0.7)	0.77
Alpha glucosidase inhibitor	28 (2.6)	7 (2.6)	6 (2.6)	9 (2.9)	6 (2.2)	0.99
Meglitinide	9 (0.8)	4 (1.5)	3 (1.3)	1 (0.3)	1 (0.4)	0.33
Insulin	309 (28.9)	61 (23.0)	58 (25.2)	85 (27.6)	105 (39.2)	**<0.01**
**ACE inhibitor/ARB (*****n**,* **%)**	609 (56.9)	148 (55.8)	120 (52.2)	193 (62.7)	148 (55.2)	0.082
**Statin (** ***n*** **, %)**	815 (76.1)	221 (83.4)	169 (73.5)	237 (76.9)	188 (70.1)	**0.003**
**Uric acid lowering agent (** ***n*** **, %)**	29 (2.7)	3 (1.1)	8 (3.5)	5 (1.6)	13 (4.9)	**0.03**

*Normally distributed continuous variables were described as mean ± SD and median (interquartile range) for non-normally distributed continuous variables, and number (%) for categorical variables*.

*Uric acid A, B, and C represent sequential inter-visit measurements*.

*ACE, angiotensin converting enzyme; ARB, angiotensin II receptor blocker; BMI, body mass index; CKD-EPI, Chronic Kidney Disease Epidemiology Collaboration; CVD, cardiovascular disease; DPP4, dipeptidyl peptidase-4; GLP-1, glucagon like peptide 1; HOMA-β, homeostasis model assessment-β-cell; HOMA-IR, homeostasis model assessment-insulin resistance; HDL, high-density lipoprotein; LDL, Low-density lipoprotein; eGFR, estimated glomerular filtration rate; NSTEMI, Non-ST-segment elevation myocardial infarction; PCI, percutaneous coronary intervention; SGLT2, Sodium glucose transporter 2; STEMI, ST-segment elevation myocardial infarction; u-ACR, urinary albumin to creatinine ratio; UA, unstable angina*.

*Bold denotes statistical significance at P < 0.05*.

### Correlations Between Uric Acid Variability Measured by ASV and Other Parameters

The ASV was positively and significantly correlated with age and creatinine levels and negatively associated with eGFR (Table 2). In turn, ASV was not significantly correlated with BMI, waist circumference, blood pressure, glycemic indices, lipid profile, and u-ACR.

**Table 2:** Correlation between uric acid variability and other parameters.

	**ASV**	
	**r**	***p*-value**
Age (years)	0.10	**0.001**
BMI (kg/m^2^)	−0.06	0.12
Waist circumference (cm) (*N* = 174)	0.078	0.30
Systolic blood pressure (mmHg)	0.26	0.58
Diastolic blood pressure (mmHg)	0.041	0.37
Duration of diabetes (years)	0.038	0.27
Fasting glucose (mg/dL)	0.017	0.58
HbA1c (%)	−0.035	0.26
HOMA-B	0.11	0.16
HOMA-IR	0.08	0.32
Total cholesterol (mg/dL)	−0.058	0.06
HDL cholesterol (mg/dL)	−0.062	0.056
LDL cholesterol (mg/dL)	−0.05	0.12
Triglyceride (mg/dL)	0.037	0.25
Creatinine (mg/dL)	0.21	**<0.01**
eGFR(CKD-EPI) (mL/min/1.73 m^2^)	−0.17	**<0.01**
u-ACR (U/g creatinine) (*N* = 298)	0.021	0.72

*ASV, average successive variability; BMI, Body mass index; CKD-EPI, Chronic Kidney Disease Epidemiology Collaboration; HOMA-β, homeostasis model assessment-β-cell; HOMA-IR, homeostasis model assessment-insulin resistance; HDL, high-density lipoprotein; LDL, Low-density lipoprotein; eGFR, estimated glomerular filtration rate; u-ACR, urinary albumin to creatinine ratio*.

*Bold denotes statistical significance at P < 0.05*.

### Risk of Cardiovascular Diseases According to Uric Acid Variability Quartile

**Figure 1:**
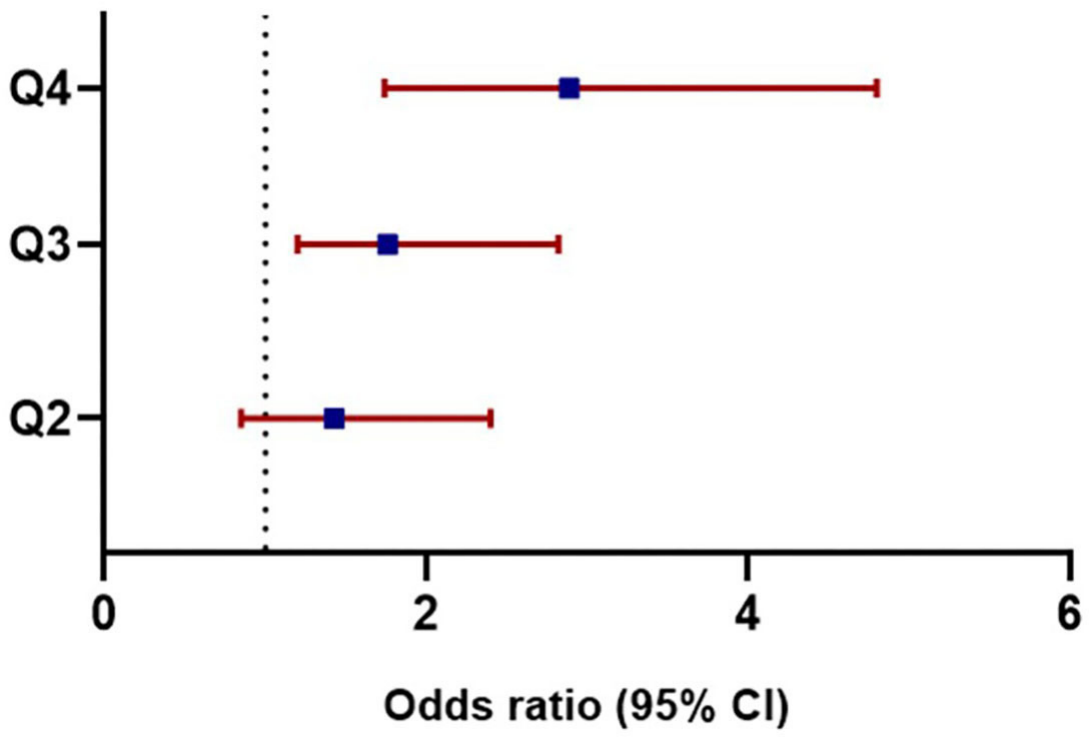
Adjusted odds ratios for cardiovascular diseases based on uric acid variability. Odds ratios in each quartile of uric acid variability increased as the quartiles advanced: Q3 (HR = 1.76, 95% CI, 1.20–2.82, *P* = 0.019), Q4 (HR = 2.89, 95% CI, 1.74–4.80, *P* < 0.001). HR, Hazard ratio; CI, Confidence interval; Q, quartile.

**Figure 2:**
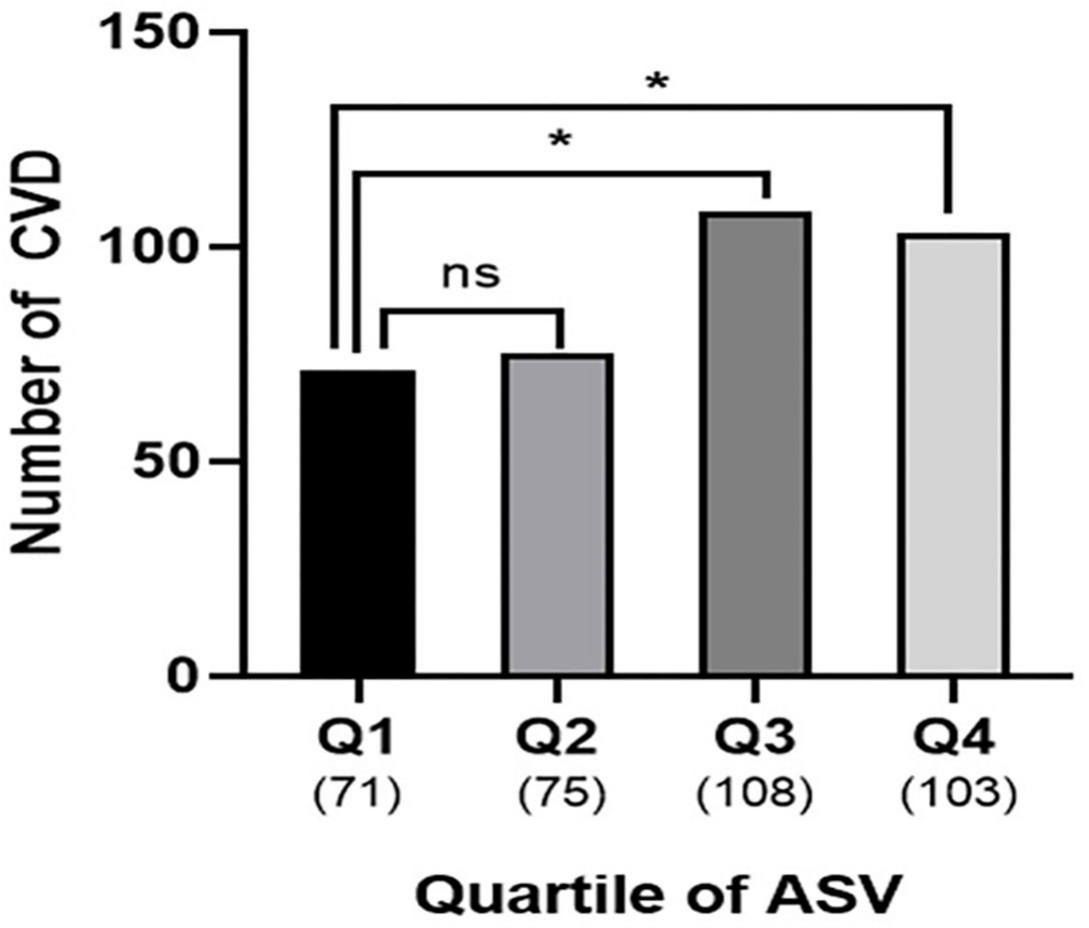
Cardiovascular outcomes according to uric acid variability. Numbers of patients with CVD were higher in quartile 3 and 4 compared to quartile 1 (Q1, *n* = 71 vs. Q3, *n* = 108 vs. Q4, *n* = 103; *P* = 0.004). Number in parenthesis represent number of CVD in each quartile. The asterisks denote the significance levels compared with Quartile 1 (control group) using chi-square test followed by *post-hoc* analyses. *P < 0.05. ns, non-specific; ASV, average successive variability; CVD, cardiovascular disease; Q, quartile.

The association between ASV and new-onset symptomatic CVD was analyzed using logistic regression analysis (Table 3). Since there was no previous study on uric acid variability and it associated parameters, the following potential confounders affecting serum uric acid were included for the analysis: age, sex, baseline uric acid, hypertension, statin use, eGFR, HbA1c, and the duration of diabetes. Even after adjusting for multiple confounding parameters in Model 4, uric acid variability increased the risk of CVD, as the quartiles increased (quartile 3 HR = 1.76; 95% CI, 1.20–2.82; *P* = 0.019; quartile 4 HR = 2.89; 95% CI, 1.74–4.80; *P* < 0.001; Table 3; Figure 1). Moreover, there was a significantly higher number of CVD events in quartiles 3 and 4 than quartile 1 (*n* = 108, 103, and 71, respectively; *P* = 0.004; Figure 2).

**Table 3:** Odds ratio for cardiovascular events by quartiles of uric acid variability.

	**Quartile 1**		**Quartile 2**		**Quartile 3**		**Quartile 4**	
	**aOR (95% CI)**	***p-*value**	**aOR (95% CI)**	***p-*value**	**aOR (95% CI)**	***p-*value**	**aOR (95% CI)**	***p-*value**
Model 1	1.00 (reference)	–	1.32 (0.90–1.95)	0.16	1.48 (1.03–2.11)	**0.034**	1.71 (1.18–2.46)	**0.004**
Model 2	1.00 (reference)	–	1.30 (0.88–1.92)	0.19	1.44 (1.00–2.07)	**0.048**	1.63 (1.12–2.38)	**0.011**
Model 3	1.00 (reference)	–	1.51 (0.99–2.29)	0.054	1.55 (1.06–2.28)	**0.025**	2.24 (1.49–3.37)	<**0.001**
Model 4	1.00 (reference)	–	1.43 (0.85–2.40)	0.18	1.76 (1.20–2.82)	**0.019**	2.89 (1.74–4.80)	<**0.001**

*Model 1 was unadjusted. Model 2 was adjusted for age, sex, and baseline uric acid. Model 3 was adjusted for Model 2 and hypertension, stain use and eGFR. Model 4 was adjusted for Model 3 and HbA1c and Duration of diabetes. Bold denotes statistical significance at P < 0.05*.

**Figure 3:**
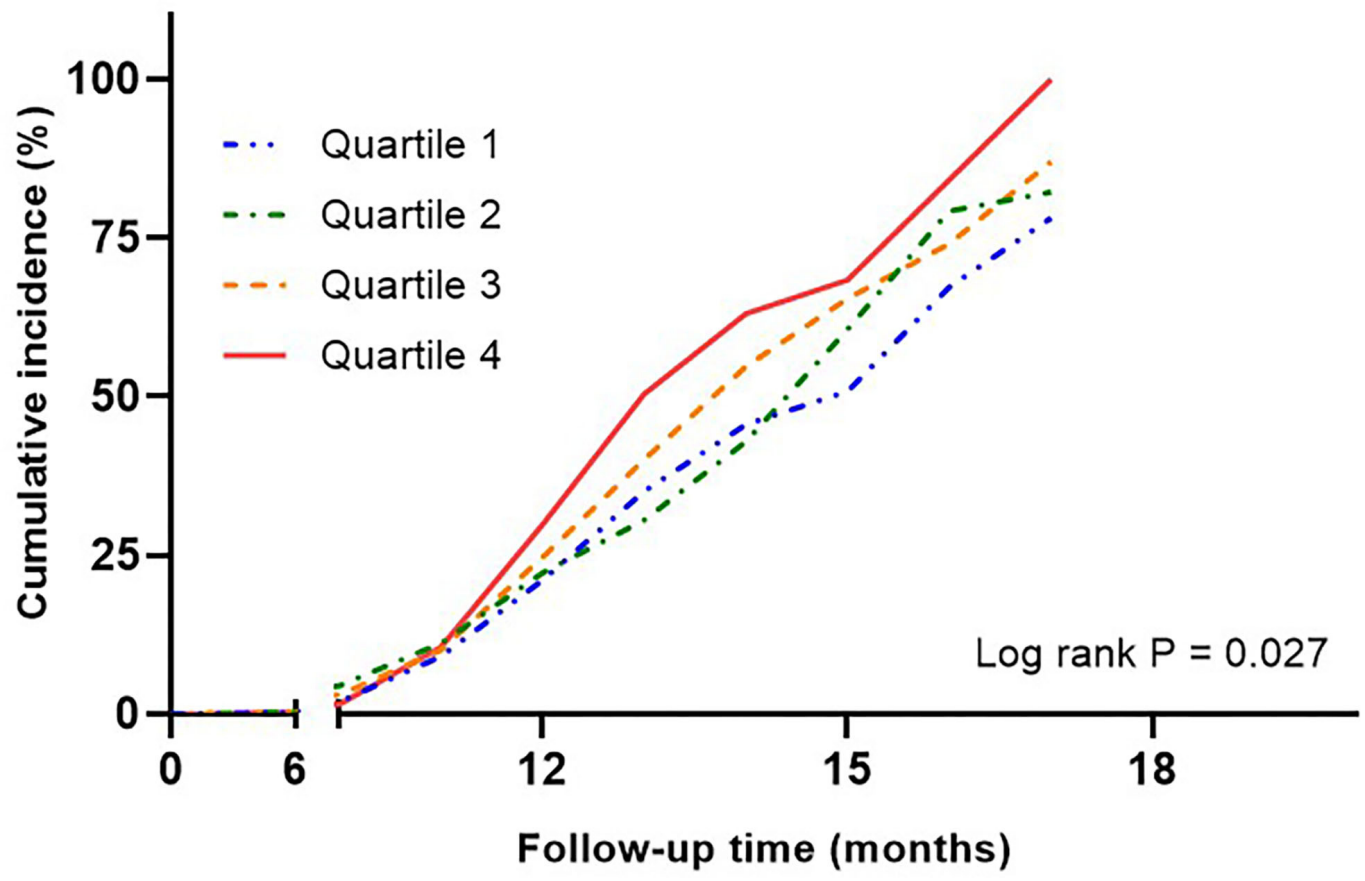
Kaplan-Meier curves assessing cumulative incidence by quartiles of uric acid variability in risk of cardiovascular disease. Adjusted for age, sex, baseline uric acid, hypertension, statin use, eGFR, HbA1c, and duration of diabetes. eGFR, estimated glomerular filtration rate.

During the median follow-up of 12 months, 357 patients had new-onset symptomatic CVD, of which 19 died. Kaplan-Meier curves showed a significant relationship between SUA quartiles and cumulative CVD events (log–rank *P* = 0.027; Figure 3). The analysis of adjusted hazard ratios showed that the risk of CVD was significantly higher in higher quartiles after adjusting for the confounders included in the multiple regression model (*p* for trend = 0.007; Supplementary Table 1). The ability of uric acid variability to predict the risk of CVD was evaluated by AUC. The AUC for ASV was 0.76, after adjusting for confounders. In previous studies, SD was most frequently used to measure uric acid variability. However, we investigated the usefulness of ASV because SDs are affected by outliers and are more appropriate for normally distributed data. The predictive accuracy of SD and ASV using a NRI and IDI was similar, with an adjusted AUC of 0.76 (pairwise comparison *P* = 0.76).

## Discussion

Uric acid is a well-established risk factor for dyslipidemia, diabetes, hypertension, coronary calcification, and renal disease (28–30) and hyperuricemia is suggested as an independent risk factor for CVD (31). Previous studies measured SUA only once in the general population. Nonetheless, there is a potential association between T2D, CVD and long-term variability of SUA (32). For this reason, we investigated the relationship between the uric acid variability and CVD in patients with T2D. This study is the first to demonstrate that increased uric acid variability is independently associated with the development of CVD in patients with T2D without previous CVD. The study population was mainly composed of older male patients with obesity who had T2D for more than 10 years, insulin-resistance and moderate glycemic control with a mean HbA1c of 7.1%. In addition, these patients presented with albuminuria and were concurrently diagnosed with hypertension and dyslipidemia. We observed that age, and creatinine significantly increased across the quartiles of uric acid variability. In contrast, there were no significant differences in glycemic and lipid profiles, except for HDL cholesterol. High uric acid variability was associated with new-onset symptomatic CVD independent of demographic characteristics, baseline comorbidities, use of medications, and glycemic status. Since the predictive accuracy of uric acid variability measured by ASV and SD was similar, higher ASV in patients with T2D are associated with an increased risk of development of new-onset symptomatic CVD.

Uric acid (2.6.8-trihydroxypurine, C_5_H_4_N_4_O_3_) is a purine derivative (33) and uric acid production depends on the rate-limiting enzymes, xanthine dehydrogenase and xanthine oxidase (XO), which are expressed predominantly in the liver, small intestine, adipose tissues, vascular endothelium, and macrophages (34, 35). XO activity determines the generation of reactive oxygen species (ROS), a major source of oxidative stress in cells (36). The major regulation of uric acid levels occurs in the kidney, where 60–70% of total body uric acid is excreted by reabsorption and secretion and the remaining uric acid effluxes into the intestine followed by bacterial uricolysis (37). High uric acid levels are associated with older age and an increase in blood pressure, cholesterol, creatinine, BMI, diuretics and alcohol use (8). Hyperinsulinemia due to insulin resistance can increase the uric acid levels by reducing renal urate and sodium reabsorption, leading to uric acid accumulation (38). Since insulin resistance is a major derivative of T2D, uric acid and its diabetogenic action are significantly correlated with risk factors for metabolic syndrome and low HDL cholesterol (30, 39).

Emerging evidences of uric acid as a marker for increased oxidative stress rather than a primary risk factor of CVD suggest that the upregulation of XO metabolic pathway leads to vasoconstriction, reduced myocardial function, oxidative stress, and hyperuricemia (40). Additionally, uric acid promotes the activation of the renin-angiotensin-aldosterone system (RAAS) (41), which is critical in the pathogenesis of CVD (42). Overall, hyperinsulinemia in patients with T2D causes incremental variations in uric acid levels over time, thus increasing oxidative stress and generating free radicals, which contribute to endothelial dysfunction and RAAS activation, ultimately leading to CVD.

Males are more associated with higher uric acid levels than females, however, uric acid levels increase in women after menopause (43) because of decreased estrogen, which is involved in renal uric acid excretion (44). Despite predominance of men in our cohort, there was no significant difference between sex and uric acid variability. The mean age of the study population was ≥60 years. Considering the average age at menopause, the women in our cohort were presumed to have passed menopause, which might explain the absence of sex differences in uric acid variability quartiles. The mean age and baseline SUA were higher as quartiles increased, consistent with previous studies (8). The proportion of the patients with eGFR <60 ml/min/1.73 m^2^ was significantly higher in the highest quartile. Our results showed a significant correlation between uric acid variability and lower eGFR in line with the literature (45). The mean u-ACR did not show significant differences in quartiles, although the patients in higher quartiles had a mean u-ACR of more than 30 U/g creatinine. Diabetic kidney disease is characterized by an eGFR of <60 ml/min/1.73m^2^ or the presence of albuminuria, which are major risk factors for developing CVD (46). The current study showed that higher quartiles of uric acid variability had a higher risk for CVD. Hence, patients with higher uric acid variability associated with a decline in eGFR or albuminuria should be screened for symptoms and signs of CVD and more stringent management of coexisting cardiovascular risk factors should be considered.

The concept of variability is applied to the control of metabolism and energy homeostasis, such as circadian rhythms (47). On the other hand, biological parameters, such as glucose and blood pressure should be maintained in a narrow ranges (48, 49). As demonstrated in a previous study (16) and the present study, the SUA levels were not pathologically high based on the definition of hyperuricemia (50). Therefore, in addition to glucose and blood pressure, SUA levels should be maintained within a narrow range to reduce the incidence of cardiovascular complications in patients with T2D. In addition, patients using urate-lowering agents were included in the study and the rate of use of these medications was higher in higher quartiles. However, the incidence of CVD was significantly higher in higher quartiles, suggesting that urate-lowering agents did not affect uric acid variability, and this parameter can be utilized to predict CVD even in patients using these medications.

No studies have assessed the correlation between uric acid variability and CVD in patients with T2D (32). A previous study explored the association between uric acid variability as measured by SD of Z-scores, and coronary heart disease and all-cause mortality in a male population without diabetes (51). Another recent study explored the association of variability in uric acid, measured by SD in patients with CVD who had previously underwent successful coronary intervention (17). Moreover, the study adjusted for diabetes to access the risk of future CVD, showing that SD was independently associated with future CVD events. However, the population was composed of patients who had previously underwent coronary intervention, which could simulate different milieu and baseline status compared to patients without previous intervention history. Thus, we adjusted for the classic risk factors of CVD and factors affecting uric acid variability and glycemic status to verify the independent association between uric acid variability and the development of new-onset symptomatic CVD in patients with T2D.

Given the lack of guidelines for proper screening and managing uric acid levels in patients with T2D, this study suggests that clinicians should closely observe uric acid variability rather than single-measured SUA in patients with T2D with or without classic CVD risk factors. For patients with high uric acid variability, appropriate diabetic medications that can decrease oxidative stress and inflammation should be prioritized, considering the proposed mechanisms of uric acid and the development of CVD. Moreover, hypertensive medications, especially ARB and SGLT2 inhibitors should be used to reduce RAAS hyperactivation in patients with high uric acid variability. Losartan increases uric acid excretion via urate transporter 1 (52) and SGLT2 inhibitors reduce SUA by the uricosuric effect secondary to glycosuria without the direct interaction of major uric acid transporters (53). The calculation of ASV is easier to perform than SD, which may facilite the assessment of uric acid variability in the clinical setting.

This study has several limitations. First, a causal relationship between uric acid variability and the development of new-onset symptomatic CVD could not be determined due to the cross-sectional study design. Second, the non-CVD group was selected by PSM. Therefore, large prospective studies with long-term follow-up are needed to validate the relationship between uric acid variability and CVD in the future. Third, the lack of adjustment for confounders of SUA including ethnicity, alcohol consumption, and use of diuretics can potentially modify the association between uric acid variability and CVD. Also, uric acid related oxidative stress markers such as C-reactive protein and tumor necrosis factor, which can affect the pathogenesis of CVD, were not measured. In this study, we presented the uric acid variability by ASV. Several other variability indices, including SD, coefficient of variation, Z-score, and variability independent of mean were calculated in this study and showed significantly higher aOR in the highest quartiles; however, none of these indices showed stepwise significance in the regression analysis. SDs are best suited to measure variability in normally distributed data (54), but ASV may be an alternative for measuring SUA in patients withT2D, where distribution of SUA could be dispersed and skewed in the research and clinical settings.

## Conclusion

Identifying risk factors for the development of CVD is essential for reducing morbidity and mortality in patients with T2D. To our knowledge, this study is first to demonstrate that increased uric acid variability is independently associated with an increased risk of CVD in patients with T2D, even in cases in which the increase in SUA was modest. Enhanced systemic pro-inflammatory responses and oxidative stress caused by fluctuation and increase of uric acid in patients with T2D may have created a milieu to develop CVD, which could explain the association between uric acid variability and new-onset symptomatic CVD. The potential use of uric acid variability as an effective marker of CVD in patients with T2D needs to be further validated in the future.

## Data Availability Statement

The datasets presented in this article are not readily available because data cannot be shared publicly due to the privacy of individuals that participated in the study. The data will be shared on reasonable request to the corresponding author. Requests to access the datasets should be directed to Eun Seok Kang, EDGO@yuhs.ac.

## Ethics Statement

The studies involving human participants were reviewed and approved by Institutional Review Board of Yonsei University College of Medicine, Seoul, Republic of Korea (Approval No. 4-2020-0610). Written informed consent for participation was not required for this study in accordance with the national legislation and the institutional requirements.

## Author Contributions

EK and HK: conception, design, analysis, and interpretation of data, drafting and revising of manuscript, and final approval of the manuscript submitted. ML, Y-hL, B-WL, and B-SC: interpretation of data, revising of manuscript, and final approval of the manuscript submitted. All authors contributed to the article and approved the submitted version.

## Conflict of Interest

The authors declare that the research was conducted in the absence of any commercial or financial relationships that could be construed as a potential conflict of interest.

## Publisher's Note

All claims expressed in this article are solely those of the authors and do not necessarily represent those of their affiliated organizations, or those of the publisher, the editors and the reviewers. Any product that may be evaluated in this article, or claim that may be made by its manufacturer, is not guaranteed or endorsed by the publisher.

## Supplementary Material

Supplementary Figure 1 Flowchart of patient selection.

Supplementary Table 1 Hazard ratio for cardiovascular events by quartiles of uric acid variability. Adjusted for age, sex, baseline uric acid, hypertension, statin use, eGFR, HbA1c, duration of diabetes. Bold denotes statistical significance at *P* < 0.05.
