# Supplementary material for: Uric Acid Variability as a Predictive Marker of Newly Developed Cardiovascular Events in Type 2 Diabetes
Source: Front Cardiovasc Med. 2021 Dec 2;8:775753. doi: 10.3389/fcvm.2021.775753 (PMC8674506; doi:10.3389/fcvm.2021.775753)
Supplement: Supplementary Figure 1 — Flowchart of patient selection. [file Presentation_1.PPTX]

## Slide 1
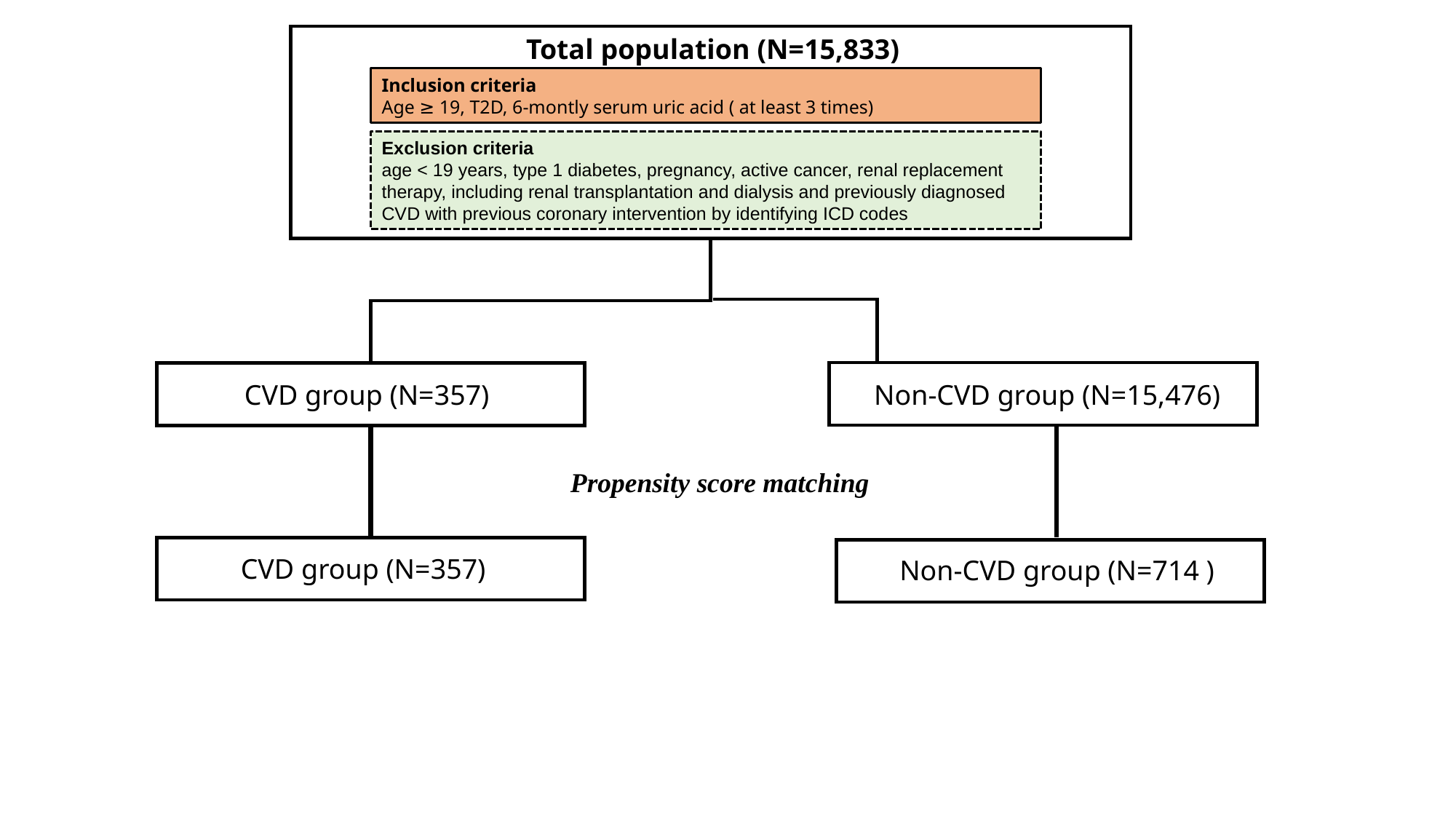

Total population (N=15,833)
Inclusion criteria
Age ≥ 19, T2D, 6-montly serum uric acid ( at least 3 times)
Exclusion criteria
age < 19 years, type 1 diabetes, pregnancy, active cancer, renal replacement therapy, including renal transplantation and dialysis and previously diagnosed CVD with previous coronary intervention by identifying ICD codes
CVD group (N=357)
Non-CVD group (N=15,476)
Propensity score matching
CVD group (N=357)
Non-CVD group (N=714 )
