# Supplementary material for: Uric Acid Variability as a Predictive Marker of Newly Developed Cardiovascular Events in Type 2 Diabetes
Source: Front Cardiovasc Med. 2021 Dec 2;8:775753. doi: 10.3389/fcvm.2021.775753 (PMC8674506; doi:10.3389/fcvm.2021.775753)
Supplement: Supplementary Table 1 — Hazard ratio for cardiovascular events by quartiles of uric acid variability. Adjusted for age, sex, baseline uric acid, hypertension, statin use, eGFR, HbA1c, duration of diabetes. Bold denotes statistical significance at P < 0.05. [file Table_1.DOCX]

**Supplementary Table 1. Hazard ratio for cardiovascular events by quartiles of uric acid variability**

|  | **Events (n)** | **Follow-up duration(months)** | **Incidence rate** | **Adjusted HR**  **(95% CI)** |
| --- | --- | --- | --- | --- |
| **CVD** |  |  |  |  |
| Quartile 1 | 71 | 12 | 5.9 | 1 (reference) |
| Quartile 2 | 75 | 12 | 6.3 | 1.20 |
| Quartile 3 | 108 | 12 | 9.0 | 1.35 |
| Quartile 4 | 103 | 12 | 8.6 | 1.92 |
| *p* for trend |  |  |  | **0.007** |

Adjusted for age, sex, baseline uric acid, hypertension, statin use, eGFR, HbA1c, duration of diabetes

Bold denotes statistical significance at *P* <0.05
